# Electronic modulation of transition metal phosphide *via* doping as efficient and pH-universal electrocatalysts for hydrogen evolution reaction†
†Electronic supplementary information (ESI) available: Additional SEM, XRD and CV curves analysis. See DOI: 10.1039/c7sc04849a


**DOI:** 10.1039/c7sc04849a

**Published:** 2018-01-04

**Authors:** Xin Xiao, Leiming Tao, Man Li, Xiaowei Lv, Dekang Huang, Xingxing Jiang, Haiping Pan, Mingkui Wang, Yan Shen

**Affiliations:** a Wuhan National Laboratory for Optoelectronics , Huazhong University of Science and Technology , Wuhan 430074 , P. R. China . Email: mingkui.wang@mail.hust.edu.cn ; Email: cica_sheny@mail.hust.edu.cn; b College of Science , Huazhong Agricultural University , Wuhan 430070 , P. R. China

## Abstract

It is highly desirable to develop efficient and low-cost catalysts to minimize the overpotential of the hydrogen evolution reaction (HER) for large-scale hydrogen production from electrochemical water splitting. Doping a foreign element into the host catalysts has been proposed as an effective approach to optimize the electronic characteristics of catalysts and thus improve their electrocatalytic performance. Herein we, for the first time, report vanadium-doped CoP on self-supported conductive carbon cloth (V-CoP/CC) as a robust HER electrocatalyst, which achieves ultra-low overpotentials of 71, 123 and 47 mV to afford a current density of 10 mA cm^–2^ in 1 M KOH, 1 M PBS and 0.5 M H_2_SO_4_ media, respectively. Meanwhile, the V-CoP/CC electrode exhibits a small Tafel slope and superior long-term stability over a wide pH range. Detailed characterizations reveal that the modulation of the electronic structure contributes to the superior HER performance of V-CoP/CC. We believe that doping engineering opens up new opportunities to improve the HER catalytic activity of transition metal phosphides through regulating their physicochemical and electrochemical properties.

## Abstract 2

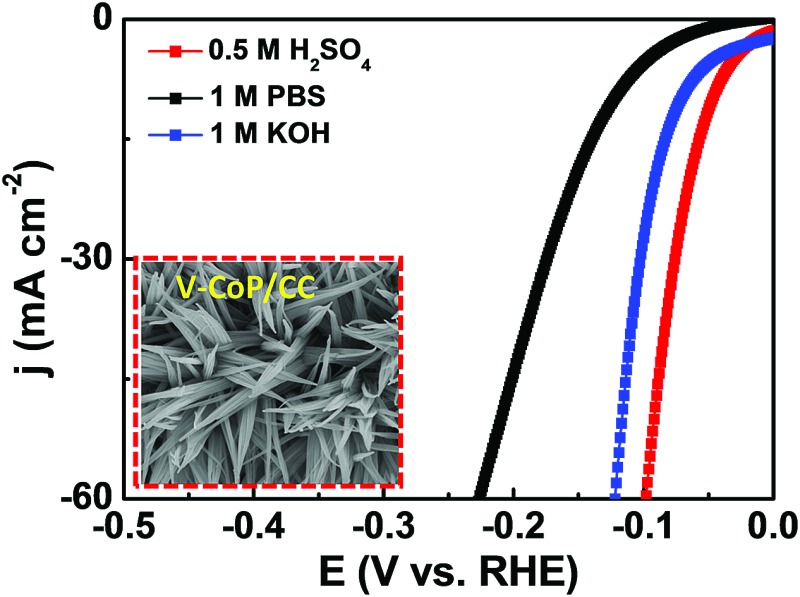
The electronic modulation of the host catalyst *via* doping provides a new strategy to efficiently boost the HER activity of transition metal phosphides.

## Introduction

The growing concerns about severe environmental pollution and rapid fossil fuel depletion have stimulated global efforts to develop renewable and sustainable energy sources. Hydrogen as an energy carrier provides a promising alternative to traditional fossil fuels due to its high energy density and zero emission of greenhouse gases.^1^–^3^ It is commonly acknowledged that the generation of hydrogen from water splitting is more environmentally friendly than through the steam methane reforming or coal gasification methods. However, the sluggish kinetics of the cathodic hydrogen evolution reaction (HER) and anodic oxygen evolution reaction (OER) in overall water splitting often require efficient electrocatalysts to reduce the electrolytic overpotential.^4^ Up to date, noble metal Pt-based materials are considered to be the state-of-the-art catalysts for the HER. However, noble metal catalysts often suffer from a high cost and limited abundance, which inevitably hinders their feasibility in large-scale production.^5^,^6^ Furthermore, the water-splitting devices based on proton exchange membrane (PEM) technology and microbial electrolysis cells (MEC) often operate in acidic and neutral media, respectively. Besides, water electrolysis in industry is usually performed in strongly basic media.^7^–^9^ Therefore, exploring highly active, cost-effective, and earth-abundant HER catalysts that can operate well over a wide pH range would be highly desirable for practical applications.

At present, transition metal-based catalysts such as chalcogenides,^10^,^11^ phosphides,^12^,^13^ carbides/nitrides,^14^,^15^ and alloys^16^,^17^ have received considerable attention due to their outstanding electrocatalytic performance. Among these materials, binary Co-based phosphides formed by alloying cobalt and phosphorus exhibit an efficient activity for hydrodesulfurization (HDS), and thus more recently have come to attention for HER due to the commonalities among catalysts for HDS and HER. Besides, the negatively charged phosphorus atoms in Co-based phosphides can efficiently capture protons due to the existence of a strong electrostatic affinity while the positively charged cobalt atoms can function as hydride-acceptor sites. These properties synergistically promote HER activity.^18^,^19^ Furthermore, transition metal phosphides exhibit a high stability for HER over a wide pH range. The above mentioned characteristics place them in the ranks of an excellent nonprecious catalyst for HER. More recently, ternary transition metal phosphides have received more attention because their electrocatalytic performance outperforms that of the corresponding binary metal phosphides, which is mainly attributed to the modulation of the electronic structure in the multimetal phosphides.^20^–^22^ Similarly, doping a foreign element into the host material could induce a change in the charge states through the occupation and energy of the anti-bonding defect levels, and thus it would be another efficient method to realize highly active catalysts for HER.^23^,^24^ Besides, very recently vanadium, a cheaper and earth-abundant transition metal element, has attracted considerable attention for application in electrocatalytic fields. For instance, Sun's group have successfully incorporated the vanadium element into Ni(OH)_2_ and the formed NiV-LDH catalyst even exceeds the best-performing non-precious NiFe-LDH material in catalyzing water oxidation.^25^ The superior catalytic activity for NiV-LDH is attributed to the enhanced conductivity, facile electron transfer, and abundant active sites, indicating the metal vanadium would be an excellent foreign dopants.

Inspired by the above discussion, we herein propose a scheme to develop an active catalyst for HER through incorporating the transition metal vanadium into two-dimensional (2D) nanoneedle arrays of cobalt phosphide. The highly active and stable electrocatalyst can be scalably synthesized *via* a hydrothermal-phosphorization method. Meanwhile, a three-dimensional (3D) conductive and flexible carbon cloth (CC) with high surface area was selected as the substrate for supporting the catalytic material. As a resultant material, the V-CoP/CC electrode requires ultra-low overpotentials of 71, 123 and 47 mV to afford a current density of 10 mA cm^–2^ in 1 M KOH, 1 M PBS and 0.5 M H_2_SO_4_ media, respectively. Meanwhile, the V-CoP/CC electrode exhibits a small Tafel slope and superior long-term stability over a wide pH range. Detailed investigation reveals that incorporation of vanadium component into CoP modulates the electronic structure of the Co electrocatalytically active center and thus further boosts the intrinsic activity of CoP.

## Experiments

### Materials

Nickel nitrate hexahydrate (Co(NO_3_)_2_·6H_2_O), vanadium chloride (VCl_3_), urea (CO(NH_2_)_2_), ammonium fluoride (NH_4_F), sodium hypophosphite monohydrate (NaH_2_PO_2_·H_2_O), potassium hydroxide (KOH), sulfuric acid (H_2_SO_4_), disodium hydrogen phosphate (Na_2_HPO_4_), sodium dihydrogen phosphate dihydrate (NaH_2_PO_4_·2H_2_O), acetone, and ethanol were all purchased from the Beijing Chemical Co. Ltd (China). The commercial Pt/C (20 wt%) catalyst and Nafion solution (5 wt%) were purchased from Johnson Matthey and Sigma-Aldrich, respectively. All the chemicals were used as-received. Deionized water (18.2 MΩ cm) was used throughout all the experiments.

### Preparation of CoP/CC and V-CoP/CC

The substrate CC (2.5 × 4 cm^2^) was cleaned sequentially in acetone, ethanol, and water solution for 15 min through sonication treatment. The preparation of V-CoP/CC nanoneedle arrays on the CC was similar to that in a reported work.^26^ In a typical synthesis, 2.7 mmol of Co(NO_3_)_2_·6H_2_O, 0.3 mmol of VCl_3_, 15 mmol of CO(NH_2_)_2_, and 9 mmol of NH_4_F were dissolved in 80 mL of deionized water under vigorous stirring. Afterwards, the cleaned CC was immersed into the solution and transferred into a Teflon-lined stainless steel autoclave, and then reacted at 120 °C for 6 h in an electric oven. After the autoclave was cooled to room temperature, the CC with the active materials was taken out and washed by water and ethanol, and finally dried at 60 °C overnight to obtain the V-Co(OH)F/CC precursor. To further prepare V-CoP/CC, the precursor V-Co(OH)F/CC and NaH_2_PO_2_ were put in a tube furnace, and then annealed at 350 °C for 120 min at a heating rate of 2 °C min^–1^ in a N_2_ atmosphere. The V_*x*_Co_1–*x*_P/CC (*x* represents the molar ratio of V and Co in the as-prepared materials, *x* = 0.05, 0.15) materials were also prepared with the same method. Meanwhile, the preparation process for CoP/CC was similar to that for V-CoP/CC except without addition of VCl_3_.

### Material characterization

All X-ray diffraction (XRD) tests were conducted on an X'pert PRO diffractometer (PANalytical B.V.) to characterize the crystal phases of the as-prepared materials. Meanwhile, the morphology and chemical element composition of the catalyst were characterized by scanning electron microscopy (SEM) and energy dispersive X-ray spectrometry (EDX), respectively, which were performed on a Nova NanoSEM 450 (FEI Company). The lattice fringes of the as-obtained materials were performed on a transmission electron microscope (TEM) using a Tecnai G2 F30 microscope. To further analyze the chemical binding states of various ions in the material, the X-ray photoelectron spectroscopy (XPS) characterizations were employed on a VG Multilab 2000. Gas chromatography (GC) analysis was performed on a GC-2020 to investigate the faradaic efficiency.

### Electrochemical measurements

All the electrochemical tests were performed in a three-electrode electrochemical cell using the CHI 660D electrochemical workstation. The carbon rod and as-prepared catalysts were used as the auxiliary electrode and the working electrode, respectively. A saturated calomel electrode (SCE) and Hg/HgO electrode were used as the reference electrodes in acid solution and alkaline solution, respectively. For a better comparison, a state-of-the-art Pt/C electrode was also prepared *via* depositing commercial Pt/C on the CC substrate. All the presented potentials in our work were converted into a commonly used reversible hydrogen electrode (RHE). The conversion formula are *E*_RHE_ = *E*_SCE_ + 0.242 + 0.0594 × pH and *E*_RHE_ = *E*_Hg/HgO_ + 0.098 + 0.0594 × pH for the SCE and Hg/HgO reference electrodes, respectively. All the polarization curves were recorded with a 95% *iR* compensation.

## Results and discussion

**Fig. 1:**
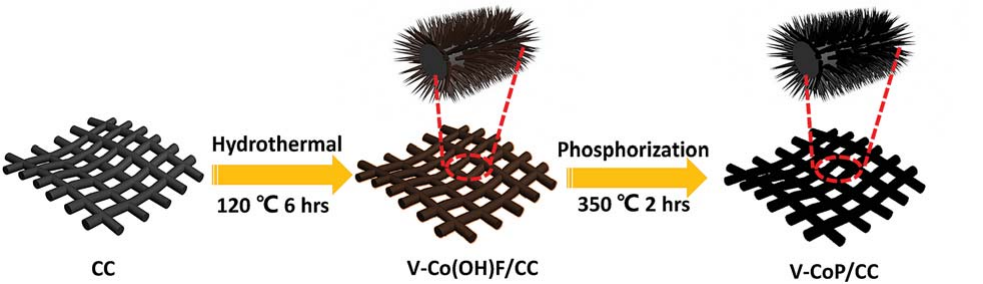
Schematic representation of V-CoP/CC preparation.

**Fig. 2:**
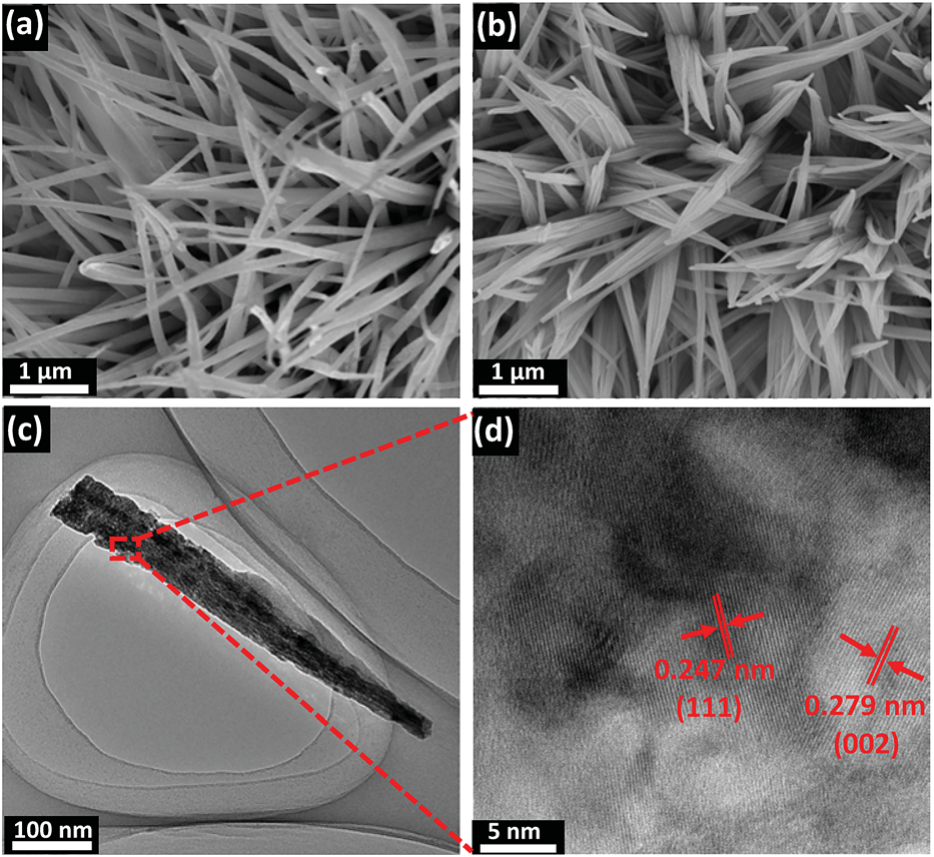
(a and b) SEM images of CoP/CC and V-CoP/CC, respectively. (c and d) HRTEM images of V-CoP/CC at different magnifications.

Fig. 1 depicts the design and integration of V-CoP nanoneedle arrays on CC to realize a high activity and cost-effective catalyst for HER. In brief, the precursor V-Co(OH)F/CC nanoneedle arrays were firstly grown on CC *via* a hydrothermal method, followed by phosphatizing the precursor V-Co(OH)F/CC using NaH_2_PO_2_ as the phosphorus source to obtain V-CoP/CC. The precursor changed from pink to light brown after the vanadium doping (Fig. S1†). Besides, the CoP/CC and V-CoP/CC both appear dark black after the process of phosphatizing. Meanwhile, scanning electron microscope (SEM) characterization shows that the nanoneedle-like arrays Co(OH)F and V-Co(OH)F (Fig. S2a and b†) are uniformly grown on CC substrates and the surface morphology shows no obvious change after a phosphorization process to form CoP and V-CoP/CC (Fig. 2a and b). However, the size of the V-CoP/CC is slightly larger than that of the pure CoP, clearly implying that the introduction of V could effectively modulate the morphology of CoP. The transmission electron microscope (TEM) image (Fig. 2c) further verified the nanoneedle-like structure of V-CoP. The lattice fringes with distances of 0.247 nm and 0.279 nm shown in high-resolution transmission electron microscope (HRTEM) images (Fig. 2d) correspond well with the d-spacing of the (111) and (002) planes of CoP, respectively.^27^,^28^ It should be noted that no lattice fringes were observed for V-based phosphides. However, the elemental mapping (Fig. S3a–d†) confirmed the existence of V element in the V-CoP/CC. The above results indicate that the V may not exist in the form of crystalline compounds, and more characterizations will be presented in the following discussion to verify this opinion. The appearance of the O element can be attributed to surface oxidation during exposure in air.^29^

**Fig. 3:**
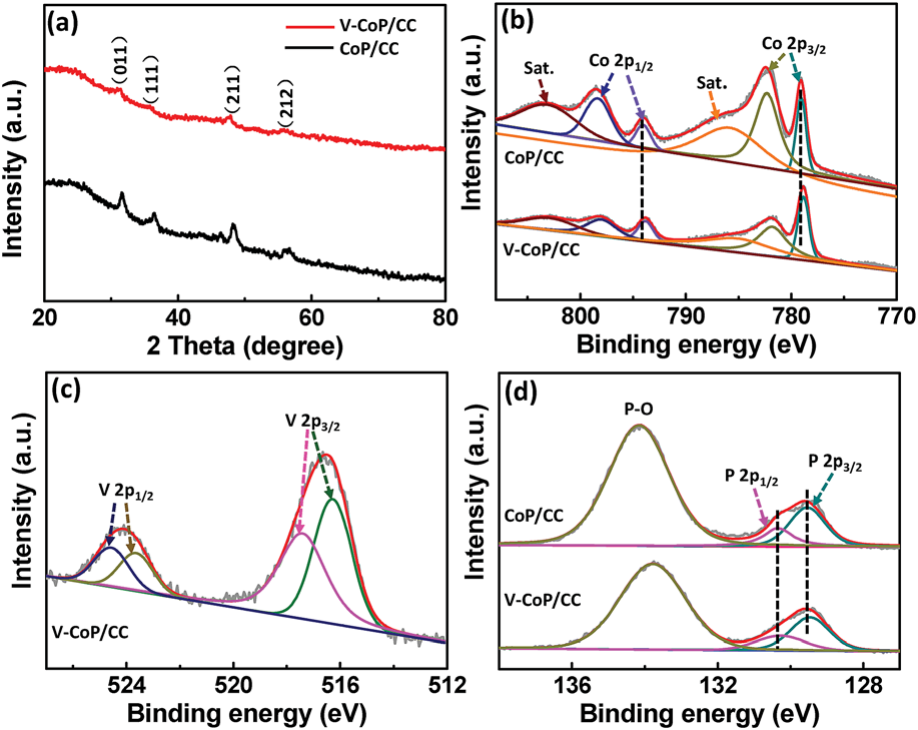
(a) XRD patterns of V-Co(OH)F/CC and V-CoP/CC. XPS spectra in the (b) Co 2p, (c) V 2p, and (d) P 2p regions for V-CoP/CC and CoP/CC.

X-ray diffraction (XRD) analysis was further applied to investigate the crystalline phase and composition of the as-prepared materials. As shown in Fig. S4a† and 3a, all the diffraction peaks for the precursor V-Co(OH)F and the corresponding phosphide V-CoP are indexed to Co(OH)F (JCPDS 50-0827) and CoP (JCPDS 29-0497), respectively,^26^ indicating that the addition of vanadium does not induce the formation of V-based compounds in our case. The intensities of diffraction peaks in a V doping system are lower than those for pure CoP, which could probably be caused by their low crystallinity. Besides, the diffraction peak at about 32° for V-CoP/CC shifts to a smaller angle compared with that for pure CoP (Fig. S4b†), indicating that the V doped CoP has a larger lattice constant than the pure CoP and, indeed, verifying that V has been doped into the crystal lattice of CoP. A Raman spectroscopy characterization was performed on the V_*x*_Co_1–*x*_P/CC (*x* = 0, 0.05, 0.1, 0.15) to further investigate the surface chemical species. As shown in Fig. S5,† all the characteristic Raman peaks for V_*x*_Co_1–*x*_P/CC (*x* = 0.05, 0.1, 0.15) are similar to those of the pure CoP. This result further confirms the formation of V doped compounds. Additionally, the introduction of vanadium probably induces Co atoms with a lower positive charge in CoP due to the metallic nature of vanadium. Hence, we accordingly studied the composition evolution of CoP and V-CoP with X-ray photoelectron spectroscopy (XPS) analysis. As shown in Fig. 3b, the binding energies (BEs) at 778.8 and 793.9 eV are assigned to Co with a partial positive charge for CoP in V-CoP/CC.^30^,^31^ The XPS spectrum for V 2p in V-CoP exhibits two pairs of peaks, which are assigned to the surface oxidized V species, such as V^4+^ (located at 516.2 and 523.7 eV) and V^5+^ (located at 517.3 and 524.8 eV), due to exposure in air (Fig. 3c).^32^ The peaks at BEs of 129.5 and 130.3 eV for P 2p in V-CoP are assigned to P with a partial negative charge in the phosphide and a BE of 133.8 eV corresponds to the phosphate due to surface oxidation (Fig. 3d).^31^ Meanwhile, the high-resolution Co 2p and P 2p spectra for pure CoP were used as a control to explore the effect of vanadium doping on the electronic structure of CoP (Fig. 3b and d). Obviously, the peaks for Co and P in V-CoP shift toward lower BEs compared with those in CoP, indicating an increase of electronic density in CoP after incorporation of the V element. This implies a strong electron interaction between Co and V in the V-CoP system. In short, above XRD, HRTEM, and XPS analysis results all demonstrate that there is no other form of crystalline V-based phosphides in the product. This implies that the as-prepared catalyst is a vanadium doped CoP compound rather than a mixture of CoP and V-based phosphides. Meanwhile, the vanadium doping exhibits a strong influence on the electronics of the host catalyst CoP.

**Fig. 4:**
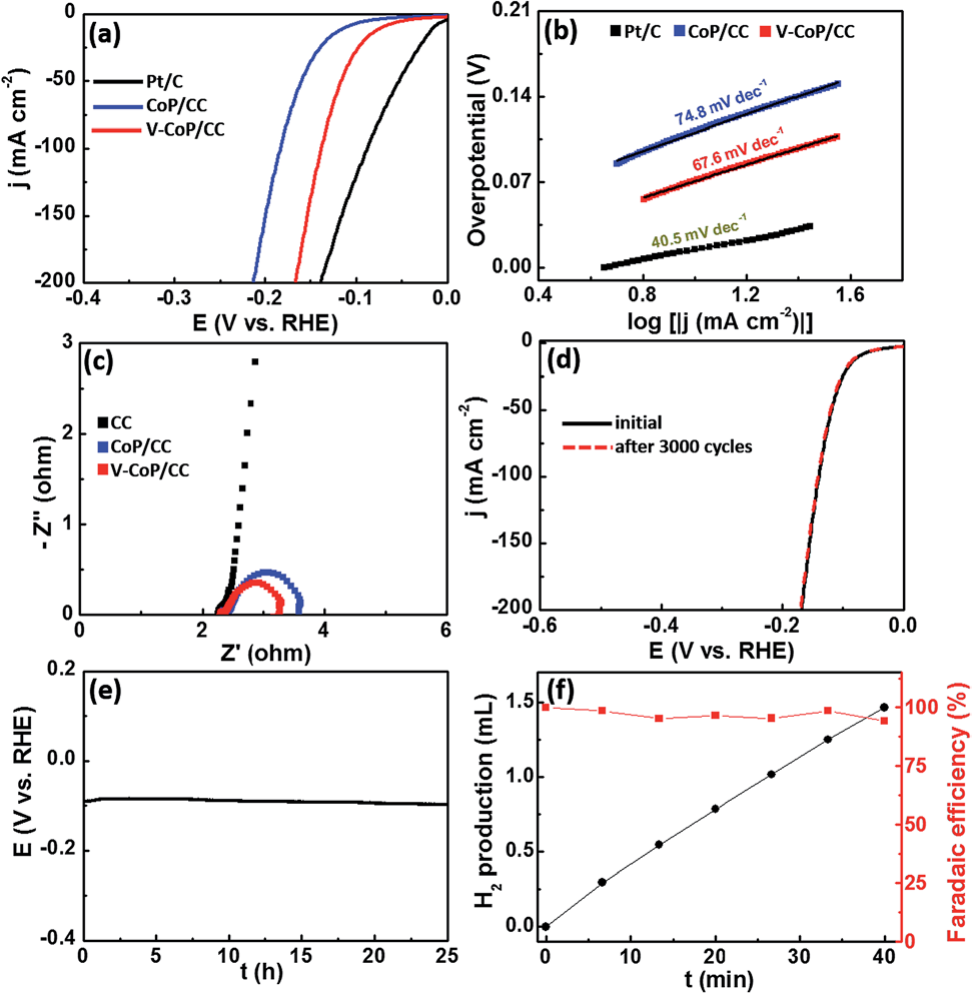
(a) HER polarization curves of bare Pt/C, CoP/CC, and V-CoP/CC in 1 M KOH. (b) The corresponding Tafel of the catalysts in 1 M KOH. (c) Nyquist plots of bare CC, CoP/CC and V-CoP/CC from 10 kHz to 0.01 Hz at –0.2 V *vs.* RHE. (d) Polarization curves of V-CoP/CC at the first cycle and after 3000 cycles. (e) The chronopotentiometric curve with a constant current density of 10 mA cm^–2^ for 25 h in 1 M KOH. (f) Faradaic efficiency (right *y* axis) and H_2_ production (left *y* axis) over a period of 40 min electrolysis at a current density of about 5 mA cm^–2^.

Considering that the strong electron interaction induced by vanadium incorporation could alter the HER kinetics on CoP, we thus evaluated the HER catalytic activity of the as-prepared samples by recording polarization curves in 1 M KOH solution. As shown in Fig. S6,† V_0.1_Co_0.9_P/CC shows the lowest overpotential among V_*x*_Co_1–*x*_P/CC catalysts (*x* = 0, 0.05, 0.1, 0.15) to afford a current density of 10 mA cm^–2^. In view of the fact that V_0.1_Co_0.9_P/CC displays the optimal catalytic performance for HER, we thus set it as the focus in the following discussion and abbreviate it as V-CoP/CC. Besides, the mass loading of the active material V_0.1_Co_0.9_P on CC substrate is 3.18 mg cm^–2^. The actual doping molar concentration of V in V_0.1_Co_0.9_P/CC is about 5.9%, obtained by an inductively coupled plasma mass spectrometry (ICP-MS) analysis (Table S1†). As shown in Fig. 4a, the overpotential required for V-CoP/CC is 71 mV to generate a current density of 10 mA cm^–2^, which is significantly lower than that for CoP/CC (112 mV). Moreover, the current density at an overpotential of 150 mV for the V-CoP/CC electrode is 137 mA cm^–2^, being about 4 times higher than that for the pure CoP/CC electrode. This demonstrates a remarkably enhanced HER activity *via* the vanadium doping in CoP. To further understand the HER mechanism, the Tafel plots were fitted with the equation *η* = *b* log(*j*) + *a* (where *η* is the overpotential, *b* is the Tafel slope, and *j* is the current density).^33^ As shown in Fig. 4b, the obtained Tafel slopes of 67.6 and 74.8 mV dec^–1^ for V-CoP/CC and CoP/CC, respectively, lie within the range of 40 to 120 mV dec^–1^. This means a Volmer–Heyrovsky mechanism for the HER on these electrode surfaces where an adsorbed hydrogen atom electrochemically reacting with a proton to produce H_2_ is the rate determining step on both electrodes. Besides, a relatively lower Tafel slope indicates a faster kinetics for V-CoP/CC, and thus a higher hydrogen generation rate was achieved on the V-CoP/CC electrode compared with the CoP/CC electrode. Moreover, the obtained exchange current density (*j*_0_) based on the intercept of the Tafel plot for V-CoP/CC was as high as 0.897 mA cm^–2^, which is about 2.7 times higher than that for CoP/CC (0.336 mA cm^–2^), indicating a faster HER kinetics on the V-CoP/CC electrode. The increased activity is probably attributed to the small charge transfer resistance.^34^,^35^ This is further confirmed by electrochemical impedance spectroscopy (EIS) measurements, showing that, indeed, the V-CoP/CC electrode has a smaller polarization resistance than that of CoP/CC (Fig. 4c). It suggests an enhanced charge transfer rate and faster catalytic kinetics on the V-CoP/CC electrode. Fig. S7† shows a multistep current polarization curve for the V-CoP/CC electrode. The potential immediately levels off at about –0.09 V at the beginning and then remains with no obvious change for the next hour. The other steps also show a similar behavior, implying good mechanical robustness, conductivity and mass transportation of the V-CoP/CC electrode.^36^

The stability is another key issue used to evaluate catalysts for practical application which is quite challenging for noble metal-free HER catalysts. To evaluate the catalyst stability in strong alkaline solution, we carried out linear scanning voltammetry (LSV) after repeated CV cycles between –0.2 and –0.1 V (*vs.* RHE). The LSV curves of V-CoP/CC before and after 3000 cycles in 1 M KOH are shown in Fig. 4d. The catalyst performs efficiently without notable loss of cathodic current density after 3000 cycles. Furthermore, the V-CoP/CC exhibits a small fluctuation in potential at a fixed current density of 10 mA cm^–2^ after a 25 h chronopotentiometric test (Fig. 4e), indicating the excellent stability of the V-CoP/CC electrode in strong alkaline media. Besides, the V-CoP/CC electrode shows about a 97% faradaic efficiency over a period of a 40 min electrolysis process at a current density of about 5 mA cm^–2^ in 1 M KOH (Fig. 4f), indicating an efficient electron transfer in the process of hydrogen generation from water splitting.

**Fig. 5:**
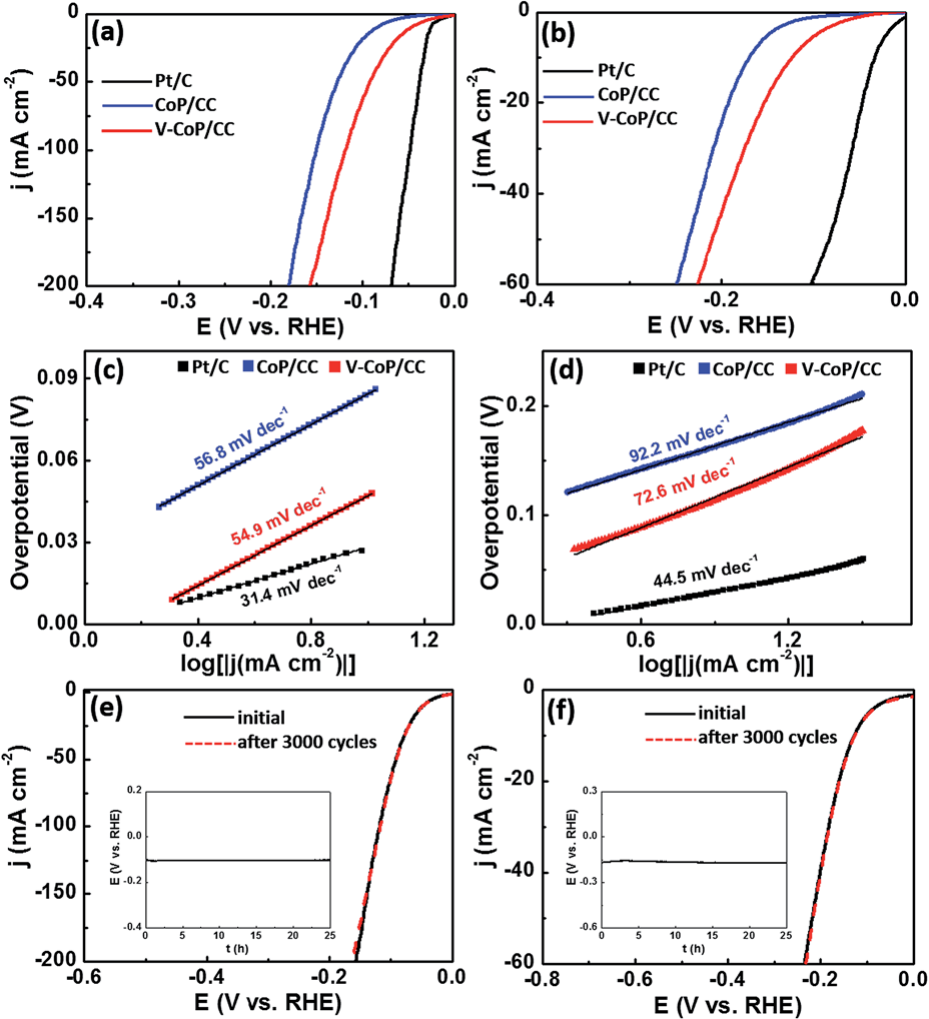
(a and b) HER polarization curves of bare Pt/C, CoP/CC, and V-CoP/CC in 0.5 M H_2_SO_4_ and 1 M PBS, respectively. (c and d) The corresponding Tafel of the catalysts in 0.5 M H_2_SO_4_ and 1 M PBS, respectively. (e and f) Polarization curves of V-CoP/CC at the first cycle and after 3000 cycles in 0.5 M H_2_SO_4_ and 1 M PBS, respectively, and the insets is the chronopotentiometric curve with a constant current density of 10 mA cm^–2^ for 25 h in 0.5 M H_2_SO_4_ and 1 M PBS, respectively.

The prepared HER catalysts that can operate at a wide pH range will certainly be of great utility due to the inevitable proton concentration change during a practical deployment. Therefore, we evaluated the HER catalytic activity of V-CoP/CC in 1 M phosphate-buffered saline (PBS, pH = 7) and 0.5 M H_2_SO_4_ (pH = 0) media. As shown in Fig. 5a and b, the V-CoP/CC electrode needs overpotentials of only 123 and 47 mV to afford a current density of 10 mA cm^–2^ in 1 M PBS and 0.5 M H_2_SO_4_, respectively. The relatively high overpotential in 1 M PBS media is probably caused by the low ion migration in PBS solution and thus results in a lower intrinsic kinetics during the process of HER.^37^ Meanwhile, the obtained Tafel slopes of 72.6 and 54.9 mV dec^–1^ for V-CoP/CC in 1 M PBS and 0.5 M H_2_SO_4_, respectively, are lower than those for the pure CoP/CC (Fig. 5c and d). This implies a higher hydrogen generation rate was achieved on V-CoP/CC. As shown in Fig. 5e and f, both polarization curves before and after the 3000 cycles test show no obvious change in 1 M PBS and 0.5 M H_2_SO_4_, respectively, and small fluctuations in overpotentials were observed as well to afford the current density of 10 mA cm^–2^ in 1 M PBS and 0.5 M H_2_SO_4_ for a 25 h stability test (insets in Fig. 5e and f). All the above discussion indicates that V-CoP/CC shows excellent catalytic activity and a high stability for HER over a wide pH range, which provides a promising electrocatalyst for practical applications.

## Conclusion

In summary, we have experimentally revealed that the electronic modulation of a transition metal phosphide through foreign metal ion doping provides a new strategy to efficiently boost the HER activity. The as-obtained V-CoP/CC catalyst exhibits an extremely low overpotential and superior long-term durability for HER over a wide pH range, which is a cost-effective alternative material for the noble metal Pt-based HER electrocatalysts. The excellent electrochemical performance of V-CoP/CC is mainly attributed to a strong electronic interaction. The material design presented in this work broadens our vision to fabricate noble-metal free catalysts for other important reactions in electrochemical energy conversion and storage.

## Conflicts of interest

There are no conflicts to declare.

## Supplementary Material

Supplementary information
