# Supplementary material for: Electronic modulation of transition metal phosphide via doping as efficient and pH-universal electrocatalysts for hydrogen evolution reaction
Source: Chem Sci. 2018 Jan 4;9(7):1970–5. doi: 10.1039/c7sc04849a (PMC5892309; doi:10.1039/c7sc04849a)
Supplement: Supplementary file 1 [file SC-009-C7SC04849A-s001.pdf]

Electronic Supplementary Material (ESI) for Chemical Science.
This journal is © The Royal Society of Chemistry 2018

Supporting Information

Electronic Modulation of Transition Metal Phosphide via Doping as Efficient and pH-universal Electrocatalysts for Hydrogen Evolution Reaction

Xin Xiao,[a] Leiming Tao,[a] Man Li,[a] Xiaowei Lv,[a] Dekang Huang,[b] Xingxing Jiang,[a] Haiping Pan,[a] Mingkui Wang,[a] Yan Shen*[a]

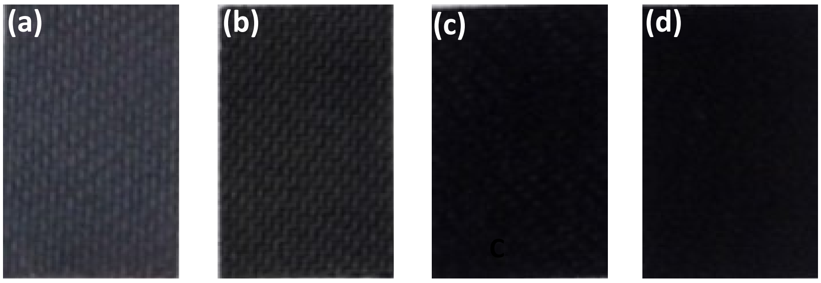

Figure S1. (a)-(d) show the photo images of Co(OH)F/CC, V-Co(OH)F/CC, CoP/CC, and V-CoP/CC, respectively.

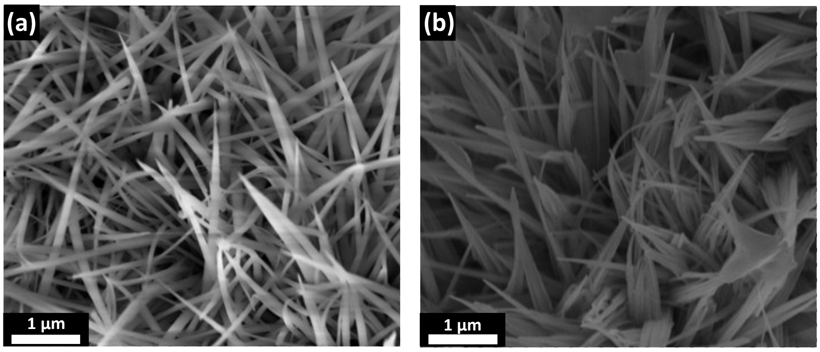

Figure S2. (a) and (b) show low- and high-magnification SEM images of Co(OH)F/CC and V-Co(OH)F/CC.

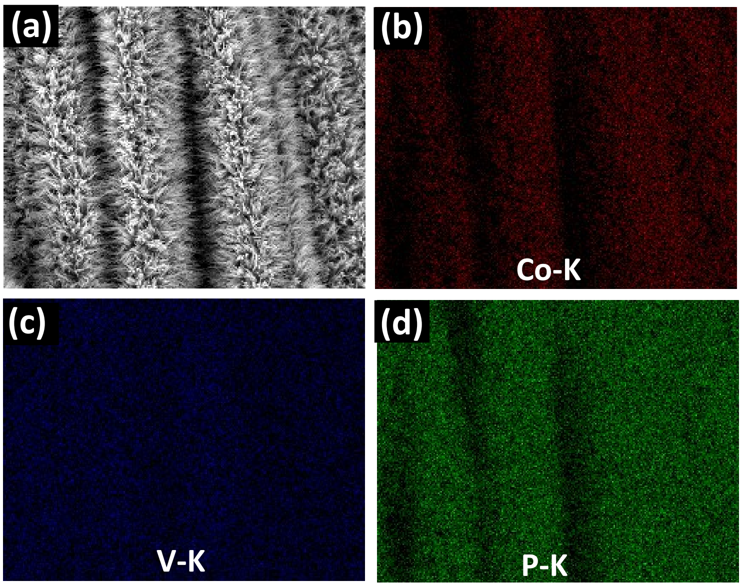

Figure S3. (a) Electron image of V-CoP/CC and the corresponding EDX elemental mapping images of (b) Co, (c) V, (d) P.

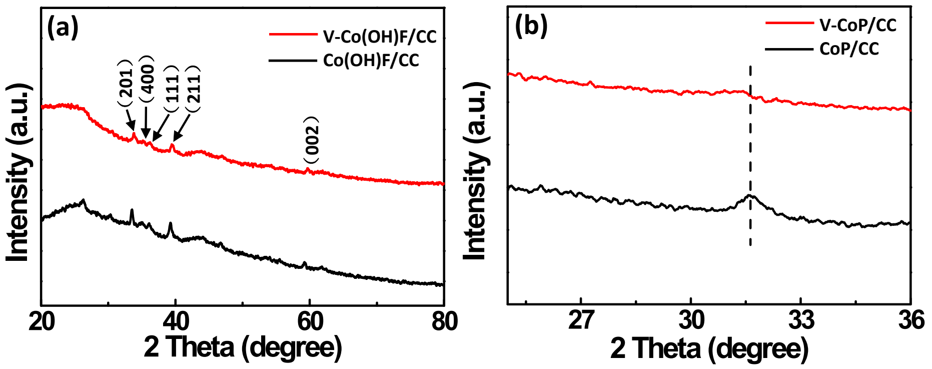

Figure S4. (a) XRD patterns of Co(OH)F/CC and V-Co(OH)F/CC. (b) The enlarged XRD patterns of CoP/CC and V-CoP/CC.

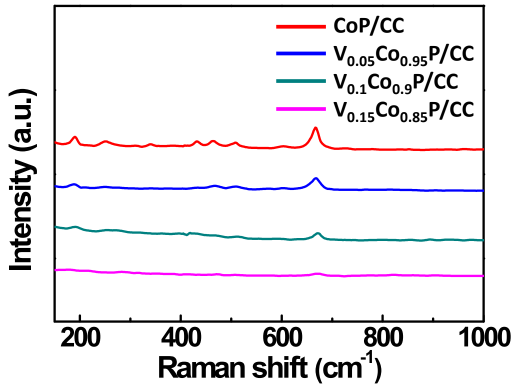

Figure S5. Raman spectra of $V_xCo_{1-x}P/CC$ (x=0, 0.05, 0.1, 0.15).

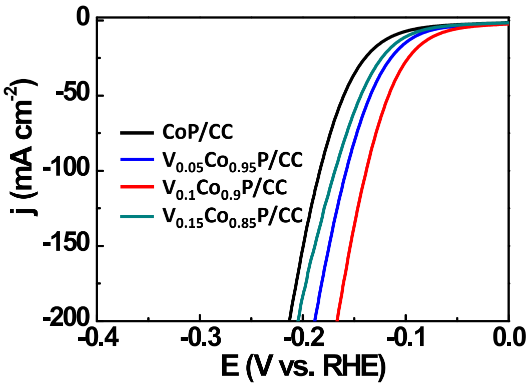

Figure S6. HER polarization curves of $V_xCo_{1-x}P/CC$ (x=0, 0.05, 0.1, 0.15) in 1 M KOH.

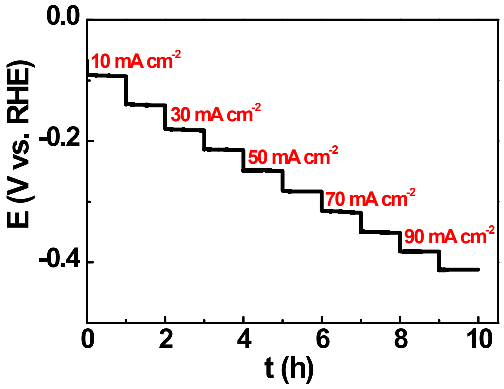

Figure S7. Multi-step chronopotentiometric curves for V-CoP/CC at varying current densities.

Table S1 Actual doping molar ratios of V in $V_xCo_{1-x}P$ analyzed by ICP-MS.

V/(V+Co)	CoP	$V_{0.05}Co_{0.95}P$	$V_{0.1}Co_{0.9}P$	$V_{0.15}Co_{0.85}P$
**theoretical value**	0	0.05	0.1	0.15
**experimental value**	0	0.024	0.059	0.093
